# Social appearance anxiety among the dark tetrad and self-concealment

**DOI:** 10.1038/s41598-024-55422-w

**Published:** 2024-02-26

**Authors:** Wenjing Jin, Tingting Zhan, Yaoguo Geng, Yibo Shi, Wanying Hu, Bei Ye

**Affiliations:** 1 School of Marxism, Zhengzhou University, Zhengzhou, 450001 China; 2 School of Education, Zhengzhou University, Zhengzhou, 450001 China; 3 Children’s Hospital Affiliated to Zhengzhou University, Zhengzhou, 450001 China

**Keywords:** Psychology, Medical research

## Abstract

This study analyzed the effects of the Dark Tetrad (narcissism, Machiavellianism, psychopathy, sadism) and self-concealment on social appearance anxiety. Empirical investigations on which personality traits influence social appearance anxiety are yet missing. In this study, a sample of N = 1186 Chinese students performed a questionnaire-based survey assessing different personality facets and social appearance anxiety tendencies. Measures included the Narcissistic Personality Inventory, the Levenson Self-Report Psychopathy Scale, the Machiavellian Personality Scale, the Short Sadistic Impulse Scale, the Self-concealment Scale, and the Social Appearance Anxiety Scale. The results of the multiple regression analysis showed that psychopathy, Machiavellianism, sadism, and self-concealment positively predicted social appearance anxiety and narcissism negatively predicted social appearance anxiety. Machiavellianism, psychopathy, sadism, and self-concealment were positive predictors of social appearance anxiety, whereas narcissism was a negative predictor. These findings provide insight into the complex nature of the Dark Tetrad and their influence on social appearance anxiety.

## Introduction

Social appearance anxiety is a special type of social anxiety caused by fear of negative evaluations of one's overall appearance by others^1,2^. In daily life, the single aesthetic appreciation praised by the media leads to a lower level of body appreciation^3^. A large number of commercial elements create an ideal body image for the public (Unified handsome men and beautiful women replace distinctive individuals) and gradually form a standardized aesthetic appreciation. The “social standard” is the aesthetic oppression of one's own body, and makes individuals change their aesthetic appreciation habits to cater to the standards^4^. When an individual's body is inconsistent with standards, it leads to the frequent occurrence of many cosmetic and plastic surgeries. Moreover, individuals who are less satisfied with their appearance tend to pay more attention to their defects, constantly comparing themselves to others. This can exacerbate anxiety and fear and ultimately lead to social appearance anxiety^5^. Social appearance anxiety is highly related to a series of disorders, such as eating disorders^6^ and social anxiety disorders^7,8^. It’s very important what kind of people are easily tempted by the ideal image created, and are more likely to have social appearance anxiety. However, little is known about possible personality predictors of social appearance anxiety. With this study, we aim to gain insight into this by investigating the effects of several undesirable personality characteristics, i.e., Dark Tetrad and self-concealment. Each of the above characters and the theoretical assumptions will be described in more detail below.

Psychopathy, narcissism, and Machiavellianism are described as the Dark Triad^9^. They are commonly associated with negative psychosocial outcomes and are considered as socially undesirable traits^10^. Machiavellianism is described as manipulativeness, selfishness, emotional coldness, and deceptiveness^11^. Narcissism is defined by a sense of entitlement, grandiosity, self-absorption, and superiority^12,13^. Psychopathy is characterized by callousness, impulsiveness, thrill-seeking, and a lack of empathy^10,14^. The Dark Triad has many problems in the process of interpersonal communication. In the interpersonal circumplex, Machiavellianism is mostly hostile-submissive, while psychopathy is a hostile-dominant interpersonal style^15^. The disturbance of interpersonal relationships and perceived social isolation make them prone to depression and anxiety. These antisocial personality traits are correlated with anxiety symptoms. Each of the Dark Triad traits appeared to be related to anxiety sensitivity^16^. More specifically, anxiety sensitivity was positively correlated with secondary psychopathy^17^. Numerous studies have shown associations were found between Machiavellianism and high levels of anxiety^18–20^. Davis found that neurotics was associated with appearance orientation, and Dark Triad was very similar to the neuroticism factor of the Big Five personality traits^21^. It showed that a high score of neuroticism was likely to mean more attention to external appearance. Previous research has found social anxiety was negatively correlated with grandiose narcissism and psychopathy, and positively correlated with vulnerable narcissism^22^. In particularly, they were associated with more emotional problems^23^. Another study also verified the relationship between specific personality traits and appearance emphasis, which showed specific personality traits were associated with the propensity of appearance management^2^. Accordingly, we hypothesized that Dark Triad, especially psychopathy and Machiavellianism, is associated with positive social appearance anxiety.

Recently, sadism has been related to the Dark Triad^24^. Sadism is the tendency to enjoy the suffering and pain of others and cruel or hostile acts for pleasure^25^. The sadistic personality predicted antisocial behavior independently of its overlap with the Dark Triad and has been proven to be effective in incremental prediction^26^. Dark Triad and sadism have overlapping and independent parts. Although some scholars believe that there is still room for improvement in the study of sadism, there is evidence to suggest that incorporating sadism into the Dark triad to form a tetrad is useful^27^. This study also helps to provide support for the extension of the Dark Triad to Dark Tetrad. In this study, sadism was included in the dark personality traits, and the relationship between sadism and the dark triad was discussed. Accordingly, we assumed sadism trait is associated with positive social appearance anxiety.

Self-concealment is defined as a predisposition to consciously conceal from others personal information that is highly intimate and negative to avoid distress^28^. Larson also took self-concealment as a personality variable to explain its role^28^. Self-concealment is associated with depression, anxiety, loneliness, alexithymia, and other negative emotions^29^. Individuals with high self-concealment will feel more pressure and pain, which may be related to their efforts to maintain a positive self-image and avoid causing others’ disgust^30^. Studies have confirmed that self-concealment is associated with higher social anxiety and negative self-evaluation^31^. Thus, we hypothesized that self-concealment is associated with positive social appearance anxiety.

Given the earlier work reviewed above, it seems reasonable to extend previous findings to the links between personality traits and social appearance anxiety. We can provide several specific contributions. First, to investigate the contribution of specific personality traits (Machiavellianism, psychopathy, narcissism, sadism, and self-concealment) to social appearance anxiety. If so, we will provide further evidence that insight into the complex nature of psychopathy, narcissism, Machiavellianism, sadism, and self-concealment, and their influence on social appearance anxiety. Second, to evaluate whether sadism adds additional variance in this context. If so, we would provide further evidence that sadism is relevant and could conclude that the Dark Tetrad is more appropriate than the Dark Triad. Third, we not only focus on the core part of Dark Tetrad but also attempt to understand its unique aspects. Consequently, it is hypothesized that different elements of the Dark Tetrad and self-concealment will have different social appearance anxiety (Machiavellianism and self-concealment may have a stronger effect on social appearance anxiety).

## Materials and methods

### Participants

To obtain a large number of participants, online questionnaires were used for data collection. Participants were recruited from a university in China. The final sample consisted of 1186 Chinese students from 15 to 25 years of age (M = 19.26, SD = 1.17), including 588 females (49.6%) and 598 males (50.4%). Among these participants, 324 (27.3%) were the only child in the family, and 612 (51.6%) were from the countryside. Students who voluntarily agreed to participate were allowed to clarify the meaning of some questions, but their responses to the items were not influenced by researchers, and all they provided informed consent for participation. Also, they were thanked and data security was adopted.

Ethical approval for the research was obtained from the institutional review board at the university before commencing the study. All procedures performed in this study involving human participants were in accordance with the ethical standards of the local institutional review board and with the 1964 Helsinki Declaration and its later amendments or comparable ethical standards.

### Measures

#### Dark tetrad

We applied the 16-item Narcissistic Personality Inventory in the Chinese version to measure narcissism (NPI-16)^32^. The scale was related to self-evaluation and behavior patterns, such as “I think I am an ordinary person” and “I don’t like to show off myself”. NPI follows a Yes or No forced-choice item-response. The total scores can be from 0 to 16 and the higher the total score, the higher the level of overt narcissism. The Cronbach’s α was 0.796 for the entire scale.

Psychopathy was measured by the Chinese 26-item version of the Levenson Self-Report Psychopathy Scale (LSRP)^33^. The LSRP captures two basic dimensions of psychopathy: Primary psychopathy and Secondary psychopathy (e.g., “People are cheated because they are stupid, and they deserve it”, “My goal in life is to possess good things as much as I can”). Agreement is rated on a 4-point Likert scale ranging from 1 (strongly disagree) to 4 (strongly agree). The Cronbach’s α was 0.872 for the entire scale.

The 16-item Chinese version of the Machiavellian Personality Scale (MPS)^34^ was used to measure the four dimensions, amorality, desire for control of Machiavellianism, desire for status, and distrust of others (e.g., “If others threaten my goal, I will deliberately undermine their efforts”, “I like to give the orders in interpersonal situations”, “Accumulating wealth is a very important goal for myself”, “To get ahead, team members always frame each other”). Agreement is rated on a 5-point Likert scale ranging from 1 (strongly disagree) to 5 (strongly agree). Cronbach’s alpha in the present study was 0.860.

Sadism was measured using the 10-item Chinese version of the Short Sadistic Impulse Scale (SSIS)^35^ to measure one dimensional sadistic traits. Example items include “I enjoy seeing people hurt”. The score of “agree/disagree” is used to measure the level of abusive impulse. The higher the score is, the higher the impulse to abuse. The Cronbach’s α was 0.786 for the entire scale.

#### Self-concealment

Self-concealment was measured using the 10-item Chinese version of the Self-concealment Scale (SCS)^36^ designed to measure the tendency to conceal personal distressing or negative information (e.g., “Some of my secrets hurt me”, “I have many things that only I know”). The agreement is rated on a 5-point Likert scale ranging from 1 (strongly disagree) to 5 (strongly agree) and higher total scores indicate greater levels of self-concealment. The Cronbach’s α was 0.931 for the entire scale.

#### Social appearance anxiety

We applied the 16-item Social Appearance Anxiety Scale in the Chinese version to measure social appearance anxiety (SAAS)^37^ rated on a 5-point Likert scale ranging from 1 (strongly disagree) to 5 (strongly agree). Example items are “I'm afraid people don't like me because of my appearance” or “I often fear that I don't meet the requirements of others for my appearance”. The total scores can reflect the level of an individual’s social appearance anxiety. The higher total scores indicate that individuals have a higher degree of social appearance anxiety. The Cronbach’s α was 0.954 for the entire scale.

### Statistical analysis

All statistical analyses were carried out using SPSS 20.0. We calculated the means, standard deviations, and reliabilities (Cronbach’s α) of the variables. The associations of social appearance anxiety and the other variables were assessed with Pearson correlations for numerical variables. We performed hierarchical regression analysis to compare the contribution of each component of the dark personality traits and self-concealment on social appearance anxiety with control variables in Step 1, Dark Triad (Machiavellianism, psychopathy, narcissism) in Step 2, sadism in Step 3, self-concealment in Step 4.

### Ethical approval

All procedures performed in studies involving human participants were in accordance with the ethical standards of the institutional review board at Zhengzhou University and with the 1964 Helsinki Declaration and its later amendments or comparable ethical standards.

### Informed consent

Informed consent was obtained from all subjects involved in the study.

## Results

### Correlations

Descriptive statistics and correlations between social appearance anxiety and the rest of the variables are shown in Table 1. We calculated bivariate correlations to see direct associations between variables. Machiavellianism shows positive correlations with psychopathy, narcissism, sadism, and self-concealment. Psychopathy correlates positively with narcissism, sadism, and self-concealment. Narcissism shows positive correlations with sadism. Sadism correlates positively with self-concealment. Social appearance anxiety shows positive correlations with Machiavellianism, psychopathy, sadism, and self-concealment, and negative correlations with narcissism.

**Table 1:** Descriptive statistics correlations for study variables.

	M	SD	1	2	3	4	5	6	7	8
1. Age	19.26	1.17	–							
2. OC	1.73	0.45	0.04	–						
3. RWP	1.17	0.39	− 0.01	0.02	–					
4. M	39.00	9.70	0.03	− 0.11**	0.08*	–				
5. P	52.85	10.26	0.06	− 0.04	0.08*	0.66**	–			
6. N	4.71	3.51	0.01	− 0.10**	− 0.08*	0.10**	0.12**	–		
7. S	1.58	1.65	0.02	− 0.04	0.05	0.07*	0.13**	0.21**	–	
8. SC	36.41	11.02	− 0.06*	− 0.04	0.11**	0.30**	0.29**	0.01	0.08*	–
9. SA	47.38	12.57	0.00	0.02	0.11**	0.24**	0.31**	− 0.06*	0.10**	0.48**

N = 1186; Sex (1 = Male, 2 = Female); RWP, Relationship with parents (1 = good, 2 = ordinary, 3 = poor); OC, Only child; P, Psychopathy; N, Narcissism; M, Machiavellianism; S, Sadism; SC, Self-concealment; SA, Social appearance anxiety; *p < 0.05, **p < 0.01.

The *t*-tests revealed a sex difference for social appearance anxiety scores, with the scores for males (*M* = 47.84, *SD* = 12.53) not significantly higher than those for females (*M* = 46.90, *SD* = 12.59, *t* = 1.29, *p* > 0.05). Scores for Machiavellianism (male: *M* = 39.98, *SD* = 10.34; female: *M* = 38.03, *SD* = 8.91;* t* = 3.44, *p* < 0.01), psychopathy (male: *M* = 55.01, *SD* = 10.51; female: *M* = 50.66, *SD* = 9.52;* t* = 7.48, *p* < 0.01), narcissism (male:* M* = 4.96, *SD* = 3.84; female: *M* = 4.46, *SD* = 3.12;* t* = 2.48, *p* < 0.05), sadism (male: *M* = 1.83, *SD* = 2.01; female: *M* = 1.33, *SD* = 1.11;* t* = 5.39, *p* < 0.01), and self-concealment (male: *M* = 37.69, *SD* = 10.64; female: *M* = 35.11, *SD* = 11.26;* t* = 4.54, *p* < 0.01) were significantly higher for males than for females.

### Regression analyses

Steps 1–4 of the regression analysis were shown in Table 2. The results showed that the Dark Triad (Step 2) explains about 10% of the variance in social appearance anxiety. The incorporation of sadism (Step 3) to the models incremented by 1% the explained variance of social appearance anxiety. The incorporation of self-concealment (Step 4) to the models incremented by 16% the explained variance of social appearance anxiety. Altogether, the predictors accounted for 27.6% of the variance in social appearance anxiety. Machiavellianism, psychopathy, narcissism, sadism, and self-concealment have their own significant incremental effects. While all effects are positive, that of narcissism is negative. Results of the hierarchical multiple regression analysis found that the Dark Triad (Machiavellianism, psychopathy) positively predicted social appearance anxiety but narcissism negatively predicted it. Sadism positively predicted social appearance anxiety. Self-concealment also positively predicted social appearance anxiety. These results largely support our hypotheses.

**Table 2:** Traits in the prediction of social appearance anxiety.

Step	*R*	*ΔR*^2^	*B*	*SE*	*β*	*t*
1						
Age	0.01	0.01*	0.00	0.07	0.00	0.02
Sex			− 0.92	0.73	− 0.04	− 1.27
OC			0.62	0.82	0.02	0.74
RWP			3.48	0.94	0.11	3.71**
2						
M	0.11	0.10**	0.10	0.05	0.08	2.07*
P			0.33	0.05	0.27	7.10**
N			− 0.31	0.10	− 0.09	− 3.05*
3						
S	0.12	0.01*	0.63	0.22	0.08	2.91*
4						
SC	0.28	0.16**	0.48	0.03	0.42	15.99**

Step 1, Age, Sex, Relationship with parents, Only child; Step 2, Dark Triad; Step 3, Sadism; Step 4, Self-concealment; *p < 0.05, **p < 0.001.

## Discussion

The present study investigated personality traits potentially predicting social appearance anxiety. Social appearance anxiety correlated positively with most of the analyzed constructs, namely Dark Triad (i.e., Machiavellianism, psychopathy), sadism, and self-concealment, but correlated negatively with narcissism. Using regression analysis, we identified the Dark Tetrad and self-concealment as the main predictors of social appearance anxiety. Machiavellianism, sadism, and narcissism further contributed to variance explanation with positive, positive, and negative incremental effects, respectively.

Results from the present study indicated that the Dark Triad contributes to social appearance anxiety. Of the “dark” personality traits, Machiavellianism and psychopathy were the main positive predictors, and narcissism was the negative predictor. This is consistent with our hypotheses, as individuals with high Machiavellianism are characterized as following self-serving strategies. They will worry that the negative evaluation from the image will damage their interests, and then produce social image anxiety. Individuals with high Machiavellianism were reported to be positively associated with impression management motives were reported to be positively associated with impression management motives^38,39^. To create and maintain a good social image, individuals with high Machiavellianism often take some strategies and measures, such as strategic pro-role behavior, to appear in good light, avoid negative reviews from others, and get available rewards^40^. Therefore, to maximize their interests, they often carry out impression management to avoid the negative evaluation of others, resulting in higher social appearance anxiety. Individuals with high psychopathy are due to impulse and lack of empathy^14^ and they are more likely to lack emotional involvement and necessary emotional effort in social interaction. These problems may make them have difficulties in interpersonal communication. Research confirmed, especially in the collective culture, that college students high in psychopathy also pursued intimacy, avoided loneliness, and relied on family and school, which made them have a greater demand for interpersonal belonging^41^. Due to the need for interpersonal belonging, they tend to pay more attention to their image and other people's evaluations of their appearance in social interactions. These can cause them to experience high levels of social appearance anxiety.

Although narcissism is characterized by overinflated and exaggerated self-importance, a high degree of uniqueness and individualism^42^, a self-aggrandizing, vanity, and flaunting personality^43^, certain components of it, such as positive self-concept and high self-efficacy^44^ can help alleviate social appearance anxiety. This may also be related to the measurement tools we used. NPI-16 cannot accurately measure grandiose narcissism and vulnerable narcissism, but can effectively measure the self-absorption and self-admiration of narcissism^45^. Consequently, narcissism was a negative predictor of social appearance anxiety in this study.

We also examined whether sadism is a relevant dark personality trait, considered an extended cluster of negative personalities—the Dark Tetrad, and added unique variance over the Dark Triad in social appearance anxiety prediction. Sadism is potentially more similar to psychopathy and Machiavellianism than narcissism^46^. Sadism improved the prediction of social appearance anxiety. Although shared features of Dark Triad and sadism (e.g., callousness and lacking empathy) could explain the findings, other explanations are credible. Sadism is related to interpersonal harm and meanness^47^, which may lead to poor interpersonal relationships. However, further research is needed to establish more solid conclusions about the unique variance of sadism in the prediction of social appearance anxiety.

Self-concealment was also associated with higher social appearance anxiety. Both self-concealment and social appearance anxiety were related to negative evaluation. Social appearance anxiety is associated with distrust of others and high awareness of interpersonal threats and malicious signals^6^. Individuals with a higher tendency of self-concealment monitor communication content and inhibit information disclosure during social^48^, and may disrupt communication and affect social anxiety. According to the theory of self-concealment, to maintain a good self-image, individuals actively suppress negative psychological experiences or traumatic experiences, and short-term inhibition can temporarily avoid other people’s negative evaluation of themselves^49^. Individuals will occupy too many psychological resources for fear of revealing their identities, bear huge psychological pressure and ideological burden, resulting in physical and mental fatigue, and not easy to communicate with others^50^. Furthermore, hiding secrets and not communicating with others will lead to loneliness, which makes teenagers more reluctant to disclose their secrets to others^51^. In short, individuals with high self-concealment tend to hide their pain because they want to show their perfect side in interpersonal communication. But these occupy more psychological resources and negative evaluation fear leads to more anxiety.

In sum, social appearance anxiety is predicted by personality traits that fear negative evaluation. The tendency to fear others’ negative evaluation (especially appearance), can lead to social appearance anxiety. However, positive self-concept and high self-efficacy can help reduce anxiety, such as narcissism.

*Several limitations of the present study should be noted. Firstly, the use of a cross-sectional design means that the study cannot establish causality between personality traits and social appearance anxiety, but can only suggest potential associations*^47^*. Secondly, the reliance on self-reported data is subject to social desirability bias. Moreover, the sample mainly comprised college students, which limits the generalizability of the findings to other populations. Thirdly, another limitation is the measure used to assess narcissism. The heterogeneity of narcissism means that existing narcissistic measurement tools may not be able to effectively identify maladaptive characteristics. The 16-item Narcissistic Personality Inventory in the Chinese version used in this study may not be able to capture all aspects of narcissism, such as grandiose narcissism and vulnerable narcissism*^45,52^*. Therefore, future studies can replicate this research using other measures of narcissism and the Dark Tetrad, including multidimensional measures, or utilize longitudinal or experimental research to explore the internal mechanisms underlying these constructs, such as self-evaluation, negative evaluation, fear, and self-esteem*.

In conclusion, the current study aimed to investigate which of these personality traits were related to social appearance anxiety. Based on the previous literature analysis, some characteristics of the relatively dark personality traits are selected for analysis. Results from the current study were consistent with the existing studies, and provide ideas for future research by studying the influence of the Dark Tetrad and self-concealment on social appearance anxiety. In this process, this study recognized the role of fear negative evaluation, which is of great significance to understanding the Dark Tetrad, self-concealment, and social appearance anxiety. The study further reveals the complexity of psychopathy, narcissism, Machiavellianism, sadism, and self-concealment, as well as their negative impact on individuals in specific situations (e.g., social appearance anxiety). Therefore, this study has a certain contribution to the study of personality and anxiety.

## Conclusions

This study is explorational research to examine the associations between personality traits and social appearance anxiety. The results of the analysis showed that (a) no sex differences in social appearance anxiety, sex differences were found in Machiavellianism, psychopathy, sadism, and self-concealment; (b) psychopathy, Machiavellianism, sadism, and self-concealment positively predicted social appearance anxiety, whereas narcissism was negative predictors. Narcissism was a protective factor against social appearance anxiety, while psychopathy, Machiavellianism, sadism, and self-concealment were associated with higher social appearance anxiety. These findings provide insight into the complex nature of dark personality traits and their influence on social appearance anxiety.

## Data Availability

All data generated or analyzed during this study are included in their entirety in this published article itself. Ethics approval, participant permissions, and all other relevant approvals were granted for this data sharing.
